# Improved drug-use patterns at 6 months post-discharge from inpatient substance use disorder treatment: results from compulsorily and voluntarily admitted patients

**DOI:** 10.1186/s12913-016-1548-6

**Published:** 2016-07-20

**Authors:** Adrian R. Pasareanu, John-Kåre Vederhus, Anne Opsal, Øistein Kristensen, Thomas Clausen

**Affiliations:** Addiction Unit, Sørlandet Hospital HF, Po. box 416, 4604 Kristiansand, Norway; University of Agder, Kristiansand, Norway; Norwegian Center for Addiction Research, University of Oslo, Oslo, Norway

**Keywords:** Substance use disorder, Compulsory treatment, Treatment outcome

## Abstract

**Background:**

Treatment services to patients with substance use disorders (SUDs), including those mandated to treatment, needs to be evaluated and evidence based. The Norwegian Municipal Health Care Act calls for mandated treatment for persons with “severe and life-threatening substance use disorder” if these individuals are not otherwise willing to be voluntarily treated and consequently risk their lives over drug use. This study aims to examine substance use–related outcomes at 6 months following inpatient treatment and to analyse factors associated with improved outcomes and abstinence.

**Method:**

This prospective study followed 202 hospitalized patients with SUD who were admitted voluntarily (VA; *n* = 137) or compulsorily (CA; *n* = 65). The European Addiction Severity Index was used at baseline and at follow-up to assess socio-demographic and substance use variables. Regression analysis was conducted to investigate factors associated with abstinence at 6 months of follow-up.

**Results:**

The frequency of use of a preferred substance showed marked improvement for both VA and CA patients (61 and 37 %, respectively) at follow-up. Seventy-five percent of VA patients using amphetamine reported improvement compared to 53 % of CA patients. At follow-up, the CA group continued to have a higher rate of injection use. The CA group had experienced higher rates of overdose in the past 6 months and lower abstinence rates (24 % versus 50 %) at follow-up. A lower severity of drug use at intake (non–injection drug use), voluntary treatment modality, and higher treatment involvement during follow-up all were significant factors associated with abstinence at 6 months after treatment.

**Conclusion:**

Voluntary treatment for SUD generally yielded better outcomes; nevertheless, we also found improved outcomes for CA patients. It is important to keep in mind that in reality, the alternative to CA treatment is no treatment at all and instead a continuation of life-threatening drug use behaviours. Our observed outcomes for CA patients support the continuation of CA treatment.

## Background

Substance use disorder (SUD) is a chronic relapsing disorder better managed by ongoing monitoring and extended services than by acute care model treatment approaches [1, 2]. Improved life functioning and quality along with substance use reduction are increasingly recognized as appropriate measures of effective addiction treatment outcomes [3].

It is generally accepted that external pressure has an influence on treatment seeking, and a high proportion of SUD patients may not have received treatment without pressure from friends, family, or the courts, which could be considered informal coercion [4]. In many countries, formal coercion is also an option when voluntary treatment has proven unsuccessful, but the compulsory hospitalization of SUD patients has been a controversial option [5]. This controversy sometimes centres on ethical or due process issues associated with use of forced entry into treatment but also often focuses on debate about the effectiveness of such compulsory treatment because motivation for change is likely to be low among those coerced into treatment [6]. Research into the effectiveness of compulsory treatment has yielded a mixed and inconclusive pattern of results, in part because of considerable differences in contexts and regulations around it [7, 8]. In Norway, the Social Services Act of 1993 allowed compulsory admissions to the hospital for persons with severe and life-threatening substance use. In 2012, this law was replaced by the Norwegian Municipal Health Care Act, in which § 10.2 (NMHCA) sanctions involuntary interventions for non-psychotic adult patients with SUDs [9]. The Act covers an option for retention (up to 3 months) when the health of the patient is seriously at risk because of extensive, prolonged substance use and voluntary efforts have proven insufficient. Despite over 20 years of practice under these compulsory treatment acts, little is known about the outcomes for the patients, as this question has not been previously addressed.

Inpatient SUD treatment is generally an effective approach that can initiate changes in behaviour and increase motivation for a lifetime of recovery rather than a situation dominated by drug use [10]. Although most of the questions of concern for inpatient treatment have been about the duration of effects, there are calls for research that examines which subpopulations of patients benefit most from various inpatient treatment modalities [11].

Assessments of such treatment effects should not only take place at treatment completion or when patients are transferred from one treatment phase to another but also after a certain time following a completed treatment episode. Especially for compulsory admitted patients (CA), it is important to examine the outcomes at some point after the initial treatment episode has ended to investigate the “real” outcomes following treatment.

### Aims

The aims of this study were to examine treatment outcomes in terms of drug use at the 6-month follow-up of inpatient SUD treatment, as follows: (1) Describe drug use and drug use patterns at the 6-month follow-up; (2) investigate changes in drug use at follow-up compared with intake to treatment, by voluntary and compulsory treatment status; and (3) analyse factors associated with abstinence and improvement in drug use at the 6-month follow-up.

## Methods

### Participants

A total of 326 SUD patients, either voluntarily admitted (VA) or CA, were invited to participate in this prospective study. Participants were eligible for this study if they were >18 years of age, had a SUD, could understand Norwegian, and were admitted at least 3 weeks prior to study inclusion, allowing them enough time for detoxification and stabilization before giving informed consent. According to the inclusion criteria, 228 were eligible, but 26 patients refused to participate. Of the 202 patients enrolled at baseline (65 CA and 137 VA), 123 (61 %) were interviewed at 6 months of follow-up. Because of limitations in funding and the large geographical uptake area, CA patients were prioritized for follow-up (82 % CA patients versus 59 % VA patients were included) because the CA patients were less represented in the sample at baseline. Thus, the higher loss to follow-up in the VA group was for mainly administrative and logistic reasons. The further attrition analysis showed no other differences between those who were and were not reached at follow-up in terms of demographic data, severity scores, or length of stay in the initial treatment episode.

### Data collection

Recruitment for this prospective study continued consecutively between January 2009 and May 2011 from three different publicly funded treatment centres in the southeastern part of Norway. SUD patients, CA or VA, were treated together in the same mixed-gender wards. The treatment wards had multidisciplinary staff, including psychiatrists, psychologists, social workers, occupational therapists, specialized nurses, and other trained staff. The centres offered treatment for patients with primary SUD, often combined with mental disorders. Treatment included assessments of somatic and mental health along with pharmacotherapy, cognitive milieu therapy, and individual motivation enhancement. Before study inclusion, the patients were either detoxified, which was verified by negative urine tests for alcohol, opioids, central stimulants (amphetamines, methamphetamines, and cocaine), benzodiazepines, and cannabis; or they spent a minimum of 3 weeks days in detoxification to establish baseline values not influenced by withdrawal symptoms.

No standard aftercare service was routinely provided by the wards themselves, but individual aftercare plans were made according to clinical needs in cooperation with primary care services or with social services. All patients were diagnosed based on a structured interview and examination in accordance with the International Classification of Diseases and Related Health Problems, 10th Revision (ICD-10). Follow-up interviews were performed 6 months after discharge from the hospitals and took place between July 2009 and December 2011, which included extensive travelling for the data collection team. Because patients came from all over the country (particularly the CA group), a team of dedicated project staff attempted to contact all patients directly by phone, mail, or post. In some cases, patients were found to be in prison or in inpatient treatment institutions, and arrangements were made to meet them there.

### Instruments and measure

The Mini International Neuropsychiatric Interview, version 5.0, was conducted at baseline to confirm the SUD diagnosis [12]. To assess demographics and severity of substance use variables, the most commonly used measure within addiction treatment research was used; the European Addiction Severity Index (EuropASI) [13, 14]. The EuropASI is a structured interview performed by trained and certified staff. Variables from the EuropASI used in the analyses included severity of substance use variables, such as frequency of substance use in the last 6 months (0 = never used; 1 = sometimes, but less than 2–3 times a month; 2 = 1–3 days a week; 3 = everyday use) and whether the patient had injection use or overdoses in the last 6 months. Whether patients were abstinent or not, was determined according to self-reported alcohol and drug use for the 30 days prior to the follow-up interview, i.e., the abstinent group had no alcohol or drug use during this period. The patients also disclosed their preferred substance of use (alcohol, amphetamine, cannabis, opioids, or benzodiazepines) and were assessed whether they had had suicidal attempts in their lifetime. Other variables used were type of admission to inpatient treatment (CA or VA).

The same questionnaire was used at follow-up. In addition, time in a controlled environment as defined by the EuropASI as days in jail or SUD treatment in the 30 days before follow-up was used.

### Analysis and statistical methods

Continuous variables are reported as means and standard deviations. Categorical variables are reported as frequencies. Changes in frequency of the preferred substance of use at the 6-month follow-up were performed using the matched-pairs Wilcoxon signed-rank test for the CA and VA patients separately. Thus, it was possible to decide whether the use of these substances had increased, decreased, or remained constant over time. Also changes in amphetamine and cannabis use are described because only these two single substances were reported as having been used as preferred substances by a sufficient number of patients to justify an analysis. Bivariate logistic regression was used to compare severity variables between groups at 6 months after treatment. Logistic regression was also used to examine predictors of abstinence at the follow-up. From bivariate analyses, variables with a *p* value < 0.2 were included in the multivariate analysis [15]. Results are presented as odds ratio (ORs) with 95 % confidence interval (CIs). *P* values < 0.05 were considered statistically significant. Analyses were performed with the Statistical Package for the Social Sciences software, version 21.0 (SPSS Inc., Chicago, IL, USA).

## Results

The 202 participants at baseline had a mean age of 30 years, and 34 % were women (Table 1). The proportion of CA patients was 32 % (65) versus 68 % (137) VA. All patients met the ICD-10 criteria for SUD; the majority had a drug use disorder (83 %). Injection use 6 months prior to hospitalization was reported by 54 % of the participants.

**Table 1:** Baseline socio-demographic variables for patients included at baseline (202) and those reached at follow-up (123) [N (%) or *mean (SD)*]

Variables	All patients, *N* = 202	Follow-up sample, *N* = 123
Mean age, years	30.0 (8.9)	30.4 (9.8)
Female	68 (34)	47 (39)
Education, years	10.8 (1.9)	10.8 (2.1)^a^
Relationship status, single^b^	136 (69)	84 (68)^c^
Main diagnosis		
Alcohol use disorder (AUD) or AUD with co- occurring drug use disorders	34 (17)	20 (16)
Drug use disorder	168 (83)	103 (84)
Severity scores		
Injection use^d^	105 (54)	71 (60)^e^
Duration of most problematic substance use, years	11.1 (7.6)	11.6 (7.6)^f^
Suicidal attempts – lifetime prevalence^g^	94 (49)	60 (51)^h^
Treatment variables		
Time in treatment, days	57 (26)	58 (26)
Compulsorily admitted	65 (32)	51 (42)

^a^ *N* = 117 ;^b^ *N* = 198; ^c^ *N* = 121; ^d^ *N* = 195; ^e^ *N* = 119; ^f^ *N* = 117; ^g^ *N* = 191; ^h^ *N* = 117

At the 6-month subsequent treatment, there was an 11 % reduction in injection use from baseline to follow-up (55 to 44 %, *p* = 0.001, Wilcoxon signed-rank test). The reductions were 10 % (from 71 to 61 %) for the CA group and 16 % (from 47 to 31 %) for the VA group.

A total of 31 CA patients (61 %) reported injecting drugs compared to 22 VA patients (31 %) (OR = 3.38, 95 % CI = 1.59–7.20, *p* = 0.001) (Table 2). Only one VA patient (1 %) reported an overdose during follow-up, compared to 11 CA patients (22 %) (OR = 19.25, 95 % CI = 2.40–154.65, *p* = 0.001). Compared to CA patients, twice as many VA patients reported total abstinence from all substances in the 30 days prior to the interview (50 % versus 24 %; OR = 0.31, 95 % CI = 0.14–0.68, *p* = 0.013).

**Table 2:** Outcome measures at 6 months of follow-up for compulsorily and voluntarily admitted SUD patients

Variable	Group	N (%)	OR^b^	95 % CI	*P* value
Patients with injection drug use (%)^a^	CA	31 (61)	3.38	1.59–7.20	0.001
	VA	22 (31)	1.00^d^		
Patients with overdose during follow-up (%)^c^	CA	11 (22)	19.25	2.40–154.65	<0.001
	VA	1 (1)	1.00 ^d^		
Patients with abstinence 30 days before follow-up (%)	CA	12 (24)	0.31	0.14–0.68	0.013
	VA	36 (50)	1.00 ^d^		

^a^ *N* = 121

^b^OR obtained from logistic regression

^c^ *N* = 122

^d^ = reference group

Both groups had a significant reduction in the frequency of the preferred substance (61 % of VA patients and 37 % of CA patients) (Table 3). Amphetamine and cannabis were significantly less used in both groups at the follow-up. However, 75 % of VA patients using amphetamine reported improvement compared to 53 % of CA patients. Improvement in cannabis use was quite similar for both CA and VA patients (62 and 61 %) (Table 3).

**Table 3:** Perceived changes in frequencies of substance use at 6 months of follow-up (from baseline)

		Mean score baseline^a^	Mean score follow-up^a^	Deterio-ration	No change	Improved	*P* value^b^
Frequency of preferred substance^c^ (*n* = 120)	VA (71)	2.7	1.5	4 (5 %)	24 (34 %)	43 (61 %)	<0.001
	CA (49)	2.8	2.2	2 (4 %)	29 (59 %)	18 (37 %)	<0.001
Cannabis^d^ (*n* = 49)	VA (28)	2.7	1.5	3 (11 %)	8 (28 %)	17 (61 %)	<0.001
	CA (21)	2.6	1.5	2 (9 %)	6 (28 %)	13 (62 %)	0.006
Amphetamine^d^ (*n* = 43)	VA (24)	2.8	1.2	0 (0 %)	6 (25 %)	18 (75 %)	<0.001
	CA (19)	2.5	1.7	2 (10 %)	7 (37 %)	10 (53 %)	0.026

^a^The ordinal ASI scale (frequency of use in the last 6 months) was defined as follows: 0 = never used; 1 = sometimes, but less than 2–3 times a month; 2 = 1–3 days a week; 3 = everyday use)

^b^ *P* value from Wilcoxon signed-rank test

^c^Preferred substance according to the ASI interview

^d^Sub-analyses of specific preferred drug if more than 40 patients reported preference for this substance

At follow-up, 48 of 123 (39 %) participants reported having abstained from all substances during the previous 30 days. However, 19 of 36 (53 %) and 7 of 12 (58 %) in the VA and CA groups, respectively, had been in a controlled environment for the majority of the 30 days before the follow-up, which for most of them meant inpatient SUD treatment. Thus, we hereafter also refer to this variable as “treatment involvement”.

Route of drug administration at intake (injection drug use), treatment modality, and treatment involvement during follow-up were all significant factors explaining abstinence at follow-up (Table 4). In this analysis we systematically included and controlled for variables that was found to be significantly different between groups at baseline [16]. The multivariate analysis retained days in a controlled environment (OR = 1.08, 95 % CI = 1.04–1.12, *p* < 0.001), non-injection use at baseline (OR = 3.36, 95 % CI = 1.32–8.56, *p* < 0.011), and voluntary admission (OR = 3.40, 95 % CI = 1.29–8.93, *p* < 0.013) as factors positively associated with abstinence at follow-up.

**Table 4:** Predictors of abstinence from baseline to follow-up (*N* = 123 patients)

Parameter	Bivariate analysis	*P* value^a^	Multivariate analysis	*P* value^b^
	OR (95 % CI)		OR (95 % CI)	
Age	1.02 (0.98–1.06)	0.267	–	
Female	1.27 (0.60–2.67)	0.528	–	
Education (years)	1.05 (0.88–1.25)	0.619	–	
Relationship status, single	0.56 (0.25–1.30)	0.175	0.45 (0.17–1.25)	0.127
Main diagnosis	1.23 (0.45–3.34)	0.687	–	
Severity scores				
Non-injection use	2.59 (1.21–5.54)	0.014	3.36 (1.32–8.56)	0.011
Years of using most problematic substance	1.01 (0.96–1.1)	0.699	–	
Overdoses	0.92 (0.43–1.97)	0.840	–	
Suicide attempts lifetime	1.32 (0.63–2.79)	0.456		
Treatment variables				
Days in treatment	1.00 (0.98–1.02)	0.511	–	
Voluntary treatment	0.31 (0.14–0.68)	0.004	3.40 (1.29–8.93)	0.011
Follow-up variables				
Abstinence at follow-up	1.51 (0.63–3.66)	0.356	–	
Time in a controlled environment (days)^c^	1.07 (1.03–1.10)	<0.001	1.08 (1.04–1.12)	<0.001

^a^ *P* value obtained from bivariate logistic regression

^b^ *P* value obtained from multivariable logistic regression; multivariable analysis included variables with *p* values <0.20 in bivariate analyses

^c^Time in controlled environment in last 30 days before follow-up interview

## Discussion

The majority of SUD patients approaching inpatient treatment engaged in injecting behaviour prior to baseline treatment and had many years of drug use experience, indicating a severe SUD level. At 6 months of follow-up, injection use showed improvement in both groups. Self-reported improvements regarding frequency of use of the “preferred substance” were observed for the majority of VA patients and more than one third of CA patients. Non-injection behaviour prior to treatment along with treatment involvement and voluntary treatment modality were positively associated with achieving abstinence at follow-up.

The SUD outcome measures (frequency of substance use, injection use, and overdoses) at 6 months of follow-up showed positive results for these SUD patients. This outcome indicates long-lasting consequences of the index treatment beyond hospitalization. Significantly more VA than CA patients reported improved outcomes. US studies have shown better outcome for CA than VA patients owing to better retention and hence longer periods in a controlled regime [17]. In contrast, comparisons of CA and VA patients in Swedish settings have shown no difference between these two groups regarding outcomes. The quality of the treatment provided seems to be a crucial factor because motivation is mutable and can be developed or diminished by the quality of support and services offered to patients, which is especially important to CA patients [18]. Structured, integrated, and long-term treatments that consider patient perspectives and needs may provide superior benefits compared to a plain “holding” strategy [17, 19].

In Norway, injection drug use is more common in CA compared to VA patients [16]. In the current work, significantly more CA than VA patients had been injecting illicit drugs at 6 months of follow-up (61 vs. 31 %). Similar high rates of injection use at follow-up have also been observed previously among injection substance users (up to 75 %) [20–22]. The continued high rate of injection use post treatment in the CA group is a challenge for treatment providers, and it is a concern that change in injection behaviour did not improve more following long-term inpatient treatment. Injection along with severe SUD provides a serious risk for overdose [23]. A long-term improvement and harm reduction for SUD patients requires addressing and changing injection behaviour, which should be an explicit goal during treatment. The time available in inpatient treatment is a window of opportunity that should be maximized in terms of prevention, intervention, testing, and vaccination for blood-borne viruses and for overdose prevention training. Specific overdose preventive programs could include aspects of identification and risk factors of overdose, recognize signs of an overdose and how to respond appropriately; call ambulance, provide rescue breathing and administer naloxone if available. Distribution of naloxone rescue kits together with overdose prevention training, to drug users prior to discharge from drug treatment would empower the drug user. This would be a way for clinicians to introduce a preventive message and harm reduction interventions prior to discharge, in a patient centered fashion.

The importance of assessing treatment outcome by principal drug of concern has been highlighted [24]. Accordingly, our results showed solid reductions in frequency of preferred drug use at follow-up compared to baseline for all patients. One explanation for this outcome might be that in Norway, the treatment for most patients in the CA group is integrated with that of the VA group, which is considered to be an approach that would benefit CA patients in particular because they then receive the treatment approaches that any other SUD patient does during treatment [25]. In addition, being in a shared environment with VA patients would likely “normalize” the treatment experience for CA patients.

In a US study, Brecht et al. performed a simple comparison between CA and VA patients in regards to different outcomes for coerced treatment for methamphetamine abuse and found no significant differences [26]. Although our study showed that the VA patients were somewhat better off at 6 months after treatment, there were marked improvements in amphetamine use also in the CA group (53 % reported reduction in the frequency of use). In terms of cannabis use, the two groups had a similar reduction; about half of both groups had reduction of use. Thus, the results suggest optimism for individuals and psychosocial intervention outcomes for SUD treatment in both VA and CA patients. In addition, having an aftercare plan and evaluating treatment outcomes in terms of appropriate patient-centred measures, such as quality of life, might become increasingly important when combinations of interventions are to be evaluated for chronic conditions within a long-term perspective [27, 28].

It has been highlighted that it remains largely unclear to what extent many of the commonly employed methods for getting people into treatment may be detrimental to the treatment process and longer-term outcomes [19]. Our results at follow-up showed a negative association between CA and abstinence when compared with VA. Nevertheless, within the CA group, we found that 24 % of patients achieved abstinence.

In the acute phase of CA treatment, the main target for the retention of patients is to provide life-saving treatment; in the longer term, the aim is to reduce drug use and increase motivation for further treatment, leading to long-term recovery [29]. Thus, our findings provide somewhat mixed results particularly regarding CA patients because many had less favourable drug use outcomes at 6 months of follow-up compared to VA patients. Still, we are optimistic that by integrating the results of our research it may help further improve abstinence rates at 6 months among CA patients.

However, the comparison between the VA and CA groups is somewhat unfair in this respect because the motivation for change was likely very different at treatment intake. Hence, the positive outcomes for the CA group need to be interpreted against this background. The only real alternative to CA treatment is no treatment at all.

### Methodological considerations

This study had some limitations that should be considered when interpreting the results. Caution should be taken in generalizing these findings on the basis of a relatively small sample at follow-up. Attrition rate at follow-up was larger in the VA group, which could have biased results toward a better outcome for the VA responders. However, the attrition analyses of background data for the VA group showed no difference between those reached at follow-up compared with non-responders. It is not ethical to randomize to voluntary treatment patients that are deemed in need for compulsory treatment. Conversely; patients that are not deemed in need for compulsory treatment should not be randomized to a CA group. Thus, there were no random allocations of the participants in this study.

This study is based on self-reported data. Although the dataset is likely representative for hospitalized SUD populations in Norway, particularly the observed outcomes for CA patients may vary considerably across settings and regions with differing laws regarding compulsory SUD treatment.

This study was, to our knowledge, the first in Norway to provide follow-up outcomes in patients hospitalized by CA with a comparison to VA patients.

## Conclusion

We showed that specialized SUD treatment provides improvement in drug use outcomes overall at 6 months of follow-up. Although voluntary treatment generally showed better outcomes, we found encouraging outcomes also among CA patients. It is important to keep in mind that the alternative to CA treatment in reality is no treatment at all and instead a continuation of life-threatening drug use behaviours. Therefore, we ideally should judge CA outcomes as contrasting with “no treatment”. Still, the results for CA patients that are comparable with those for VA treatment provide support for the continuation of CA treatment.

## Abbreviations

CA, compulsory admitted; CI, confidence intervals; EuropASI, European addiction severity index; ICD-10, International classification of diseases and related health problems; NMHCA, Norwegian Municipal Health Care Act; OR, odd ratio; SUD, substance use disorders; VA, voluntarily admitted
